# Intensive care outcomes in bone marrow transplant recipients: a population-based cohort analysis

**DOI:** 10.1186/cc6923

**Published:** 2008-06-11

**Authors:** Damon C Scales, Deva Thiruchelvam, Alexander Kiss, William J Sibbald, Donald A Redelmeier

**Affiliations:** 1 Department of Critical Care, Sunnybrook Health Sciences Centre, 2075 Bayview Avenue, Room D108, Toronto, Ontario, Canada, M4N 3M5; 2 Interdepartmental Division of Critical Care, University of Toronto, 30 Bond Street, Queen Street Wing, Room 4-042, Toronto, Ontario, Canada, M5B 1W8; 3 Institute for Clinical Evaluative Sciences, 2075 Bayview Avenue, Room G106, Toronto, Ontario, Canada, M4N 3M5; 4 Department of Medicine, Sunnybrook Health Sciences Centre, 2075 Bayview Avenue, Room D474, Toronto, Ontario, Canada, M4N 3M5

## Abstract

**Introduction:**

Intensive care unit (ICU) admission for bone marrow transplant recipients immediately following transplantation is an ominous event, yet the survival of these patients with subsequent ICU admissions is unknown. Our objective was to determine the long-term outcome of bone marrow transplant recipients admitted to an ICU during subsequent hospitalizations.

**Methods:**

We conducted a population-based cohort analysis of all adult bone marrow transplant recipients who received subsequent ICU care in Ontario, Canada from 1 January 1992 to 31 March 2002. The primary endpoint was mortality at 1 year.

**Results:**

A total of 2,653 patients received bone marrow transplantation; 504 of which received ICU care during a subsequent hospitalization. Patients receiving any major procedure during their ICU stay had higher 1-year mortality than those patients who received no ICU procedure (87% versus 44%, *P *< 0.0001). Death rates at 1 year were highest for those receiving mechanical ventilation (87%), pulmonary artery catheterization (91%), or hemodialysis (94%). In combination, the strongest independent predictors of death at 1 year were mechanical ventilation (odds ratio, 7.4; 95% confidence interval, 4.8 to 11.4) and hemodialysis (odds ratio, 8.7; 95% confidence interval, 2.1 to 36.7), yet no combination of procedures uniformly predicted 100% mortality.

**Conclusion:**

The prognosis of bone marrow transplant recipients receiving ICU care during subsequent hospitalizations is very poor but should not be considered futile.

## Introduction

Bone marrow transplantation is a heroic element of therapy for leukemia, lymphoma, and some other devastating diseases. The procedure sometimes yields improved long-term survival, yet it can entail significant morbidity during the initial recovery [1-3]. About 40% of patients receive intensive care unit (ICU) treatment with the initial transplant [4]. The specific reasons for ICU admission frequently involve pulmonary, hepatic, or neurological dysfunction [5-8]. In addition to monitoring techniques such as continuous blood pressure recording, ICU care often involves complicated treatment including mechanical ventilation, renal replacement therapy, and continuous medication infusions.

The utility of expensive ICU treatments for bone marrow transplant recipients is uncertain (Additional File 1). Two studies recruited patients prospectively [9,10], whereas most past research is based on retrospective cohort studies. Only two prior studies involved multiple centers [9,11]. All studies have concentrated on short-term outcomes following ICU admission during the early post-transplant period. Most studies of patient outcomes following ICU admission in bone marrow transplant recipients measured only hospital mortality, although five studies reported 6-month survival [12-16] and one study reported 1-year survival [11]. Almost all prior studies have a small sample size.

Authorities suggest that ICU admission following bone marrow transplantation is associated with a poor prognosis [17,18]. No study, however, has examined whether the poor prognosis extends to subsequent hospitalizations. Furthermore, greater knowledge of long-term outcomes would be useful to policymakers, ethicists, and other stakeholders [19,20]. The universal healthcare system in Ontario provides a unique opportunity to study long-term outcomes of unusual conditions across multiple study centers and for an entire population. We therefore used the Ontario health databases to evaluate survival of bone marrow transplant patients admitted to the ICU. Whereas previous research focused on outcomes of patients requiring ICU at the time of transplant, we examined the ICU stay during subsequent hospital admissions.

## Methods

### Identification of bone marrow transplant

We identified all adults (age > 18 years) who underwent bone marrow transplant in the province of Ontario using the Ontario Health Insurance Plan database. This database contains fee-for-service claims for services provided by physicians to Ontario residents [21,22]. The study period spanned 1 January 1992 to 31 March 2002, representing all years for which data were available. There were no exclusion criteria for the present study.

Bone marrow transplant recipients were linked to the Canadian Institute for Health Information Discharge Abstract Database, which contains demographic data, administrative data, and clinical data for hospital discharges and day surgeries in Canada. Individuals were also linked to the Registered Persons Database, which contains vital statistics on Ontario citizens. These databases have been used extensively in past research [23-26].

The admission containing the most recent discharge date was retained when multiple records had the same unique patient identifier, admission date, and date of birth. If multiple bone marrow transplants were performed on the same patient, we only considered the first procedure. If duplicate records were identical for unique patient identifier and admission date, the record associated with the most recent discharge date was retained. If discharge dates were also identical, one of the records was randomly deleted.

### Identification of subsequent ICU admission

We identified admissions to the ICU using codes in the Ontario Health Insurance Plan database according to a previously described algorithm [27]. The Ontario Health Insurance Plan database contains all claims submitted to the single-payer healthcare system for reimbursement for physician services. We focused on the first ICU admission following the hospital discharge for bone marrow transplantation. When ICU codes were interrupted by >1 day, we assumed the patient had been discharged and then readmitted to the ICU. We identified ICU admissions diagnosed as a complication of bone marrow transplant using the corresponding International Classification of Diseases, Ninth Revision, Clinical Modification Code (996.8); this code does not distinguish between graft versus host disease and other complications of bone marrow transplant. Similarly, we identified the diagnoses of acute renal failure (Codes 584.5, 584.6, 584.7, 584.8 and 584.9) and acute hepatic failure (Codes 570, 573.3, 572.2, 572.4, 782.4 and 286.7) during the hospital stay using previously described code combinations [28,29].

We identified common procedures using codes available in the Ontario Health Insurance Plan database. Mechanical ventilation was identified using a previously described algorithm [27]. We defined pulmonary artery catheter use by the presence of the specific code for this procedure during the ICU admission. Similarly, patients who had codes for acute hemodialysis were defined as receiving renal replacement therapy.

### Analysis

The primary outcome was mortality at 1 year. The end of the observation period was 31 March 2003, so that all patients were followed for at least 1 year. We analyzed the frequency of death using the chi-square test. Odds ratios and 95% confidence intervals (CIs) were estimated using univariate logistic regression. We decided in advance to stratify patients into categories based on the following characteristics: autologous versus allogeneic bone marrow transplant; need for mechanical ventilation; provision of mechanical ventilation for >10 days; receipt of hemodialysis; and insertion of a pulmonary artery catheter.

Life tables were constructed to create Kaplan–Meier curves for survival and for ICU-free survival. For the Kaplan–Meier analysis of ICU-free survival, we considered the event to be admission to the ICU following hospitalization for bone marrow transplant. Patients were censored at the end of follow up or at death. All *P *values were two-tailed and analyses were conducted using SAS software (version 9.13; SAS Institute Inc., Cary, NC, USA).

### Ethics

The need for informed consent was waived for this analysis of administrative health data. The study was approved by the ethics committee of the Sunnybrook Health Sciences Centre and was conducted using confidentiality safeguards at the Institute for Clinical Evaluative Sciences in Ontario.

## Results

**Figure 1:**
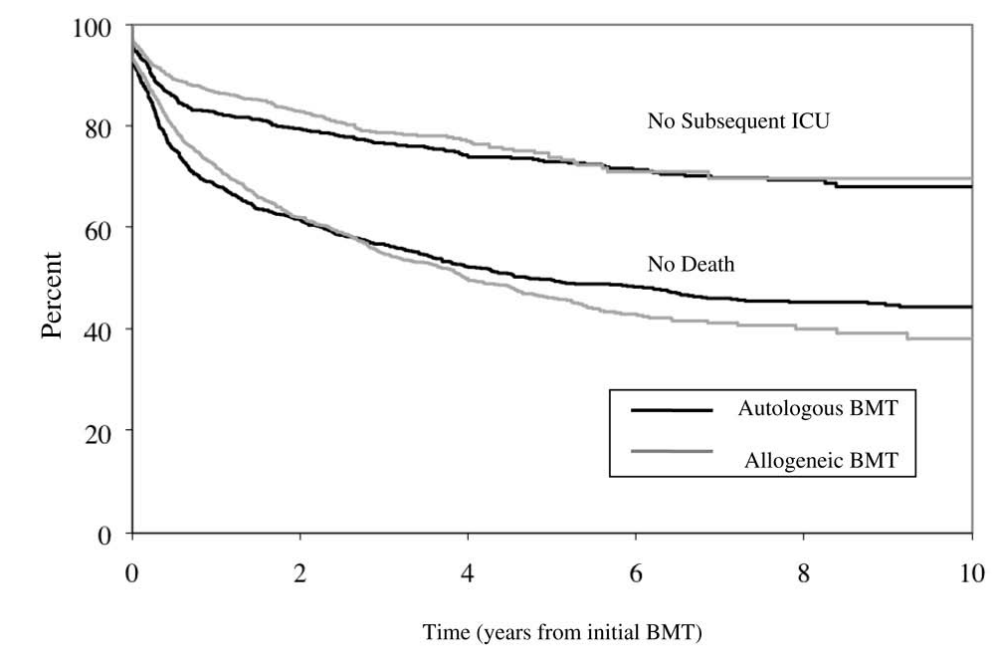
**Time from bone marrow transplant to intensive care unit admission**. Kaplan–Meier curves showing outcomes following bone marrow transplant (BMT) hospitalization. *y *axis, percentage of original cohort remaining event-free following discharge from BMT (n = 2,653); *x *axis, time in years from BMT discharge. Curves represent patients still alive following BMT hospitalization (no deaths, lower curves) and patients remaining free of the intensive care unit (ICU) following BMT hospitalization (censoring both deaths and patients lost to follow up) (no subsequent ICU, upper curves). Black lines, survival following autologous BMT; gray lines, survival following allogeneic BMT.

We identified 2,653 patients who underwent a first bone marrow transplant during the study, of whom 60% received allogeneic transplants (Table 1). The underlying diagnosis was malignancy in most cases. Almost all of the procedures (n = 2,631; 99%) were performed at seven different centers (range, 45 to 1,543 transplants per center), and no association was apparent between 1-year mortality and the bone marrow transplant procedure volume (Spearman's rank correlation ρ = 0.14, *P *= 0.76). Only 175 (6.6%) patients died during the initial bone marrow transplant hospitalization. On average, survivors required hospitalization 1.2 times (median, 1.0; interquartile range, 0 to 2) during the first year following transplant and 2.2 times during the entire study period (median, 1.0; range, 0 to 20; interquartile range, 0 to 3). During these subsequent hospital admissions, 504 (20%) patients received ICU care – typically (351 patients; 70%) during the first year following the original transplant procedure (Figure 1).

**Table 1:** Characteristics of patients with and without intensive care unit admission following bone marrow transplant hospitalization

	All patients (n = 2,653)	No ICU admission following BMT hospitalization (n = 1,974)^a^	ICU admission during subsequent hospitalizations (n = 504)	*P *value^b^
Age (standard deviation) at BMT (years)	44 (12)	44 (12)	43 (11)	0.04
Female gender (%)	1,221(46)	907 (46)	217 (43)	0.24
Allogeneic BMT (%)	1,583(60)	1,215 (62)	264 (52)	0.0002
ICU stay during BMT admission (%)	2,544(96)	1,877 (95)	496 (98)	0.013
Mechanical ventilation during BMT admission (%)	181 (7.1)	31 (2.0)	54 (11)	<0.0001
Length of hospital stay (standard deviation) during BMT admission (days)	32 (21)	31 (20)	33 (19)	0.0028
Physician claims (standard deviation) during 3 years preceding BMT admission	151 (83)	145 (74)	172 (107)	<0.0001
Charlson score > 2 during BMT hospitalization (%)	150 (5.6)	128 (6.0)	12 (2.0)	0.0004
Malignancy	2,582(97)	1,920 (97)	492 (98)	0.66
Leukemia	948 (36)	620 (31)	227 (45)	<0.0001^c^
Lymphoma	831 (31)	666 (34)	125 (25)	
Multiple myeloma	461 (17)	364 (18)	86 (17)	
Breast cancer	151 (5.7)	131 (6.6)	15 (3.0)	

^a^Excludes the 175 patients who died during bone marrow transplant (BMT) admission.

^b^For comparison of patients with versus patients without intensive care unit (ICU) admission following bone marrow transplantation hospitalization.

^c^For comparison of patients with versus patients without ICU admission following bone marrow transplantation across subtypes of malignancy.

The median time from the original transplant discharge to subsequent ICU admission was 124 days (interquartile range, 24 to 584 days). The frequencies of ICU admission comparing autologous bone marrow transplant recipients with allogeneic recipients were similar in the long run (Figure 1). The mean age was 43 years (standard deviation, 11 years), and the median length of the first ICU stay was 4 days (interquartile range, 2 to 10 days). The main reasons for hospital admission varied but included infection (n = 82, 16%), respiratory failure (n = 72, 14%), and cardiac failure (49 patients, 9.7%) (Table 2). One-third of patients (n = 154) were admitted with a complication of the bone marrow transplant (including graft versus host disease), although this was rarely the most responsible diagnosis (n = 30). Acute renal failure developed in about one in five patients (n = 97), whereas acute hepatic failure was rare (n = 17). Few of the patients (n = 66) received a second bone marrow transplant during this subsequent hospitalization.

**Table 2:** Most responsible diagnosis for bone marrow transplant recipients requiring intensive care unit during subsequent hospitalizations

Most responsible diagnosis	n (%)
Hematological/lymphatic malignancy	186 (37)
Infection^a^	82 (16)
Respiratory failure	72 (14)
Cardiac disease	49 (9.7)
Complication of bone marrow transplant	30 (5.9)
Hematologic abnormality	19 (3.7)
Gastrointestinal disorder	16 (3.2)
Solid tumor	11 (2.2)
Renal disorder	7 (1.4)
Other	17 (3.4)

^a^Includes pneumonia.

### Specific procedures

Mechanical ventilation at any time during subsequent hospitalizations was provided to about one-half (n = 258) of 504 bone marrow transplant recipients admitted to the ICU, but was rarely continued for >10 days (n = 67; Table 3). Hemodialysis during the ICU stay was provided to 35 (6.9%) patients, and pulmonary artery catheterization to 94 (19%) patients. Multiple procedures were frequently performed; for example, 86 (33%) mechanically ventilated patients also received pulmonary artery catheterization and 29 (11%) mechanically ventilated patients also received hemodialysis.

**Table 3:** Bone marrow transplant recipients requiring mechanical ventilation during subsequent hospitalizations

	Died^a ^(n = 224)	Survived (n = 34)
Demographics		
Mean (standard deviation) age (years)	43 (12)	44 (10)
Female sex (%)	45	26
Type of bone marrow transplant		
Allogeneic transplant (%)	48	59
Autologous transplant (%)	52	41
Intensive care unit procedures		
Mechanical ventilation > 10 days (%)	28	15
Pulmonary artery catheterization (%)	35	21
Hemodialysis (%)	12	6

^a^Died within 1 year of intensive care unit admission

### Outcomes following ICU admission

Mortality 1 year following ICU admission (Table 4) was not significantly related to the type of transplant (autologous 70% versus allogeneic 66%, *P *= 0.33) and was similar comparing early study years (January 1992 to March 1998, 69%) with later years (April 1998 to March 2002, 66%; *P *= 0.59). Longer intervals between bone marrow transplant and ICU admission decreased the 1-year risk of dying (odds ratio, 0.73; 95% confidence interval, 0.65 to 0.83) for each additional year between hospitalizations (*P *< 0.0001). The mean years from transplant to subsequent ICU admission was two times longer for survivors than for decedents (1.6 versus 0.8, *P *< 0.0001). Patients admitted to hospital for complications of the bone marrow transplant (including graft versus host disease) had particularly high mortality at 1 year (81%; 95% confidence interval, 73% to 86%). As expected, mortality at 1 year was also high for patients who developed acute renal failure (89%; 95% confidence interval, 81% to 94%) or acute hepatic failure (71%; 95% confidence interval, 44% to 90%).

**Table 4:** Outcomes of bone marrow transplant recipients admitted to the intensive care unit

	All ICU patients		Mechanical ventilation		Pulmonary artery catheter		Hemodialysis	
	
	Number (%^a^)	Died^b ^(%)	Number (%)	Died (%)	Number (%)	Died (%)	Number (%)	Died (%)
BMT special care unit during first BMT hospitalization	2,544 (96)	734 (29)	181 (7.1)	146 (81)	74 (2.9)	65 (88)	73 (2.9)	51 (70)
ICU within first year after BMT hospitalization	351 (13)	261 (74)	202 (58)	183 (91)	78 (22)	74 (95)	24 (6.8)	23 (96)
ICU within first 3 years after BMT hospitalization	450 (17)	317 (70)	241 (54)	214 (89)	86 (19)	81 (94)	29 (6.4)	28 (97)
ICU anytime during study period after BMT hospitalization	504 (19)	340 (67)	258 (51)	224 (87)	94 (19)	86 (91)	35 (6.9)	33 (94)

^a^Percentage of total (n = 2,653) bone marrow transplant (BMT) patients.

^b^Died within 1 year of intensive care unit (ICU) admission.

**Figure 2:**
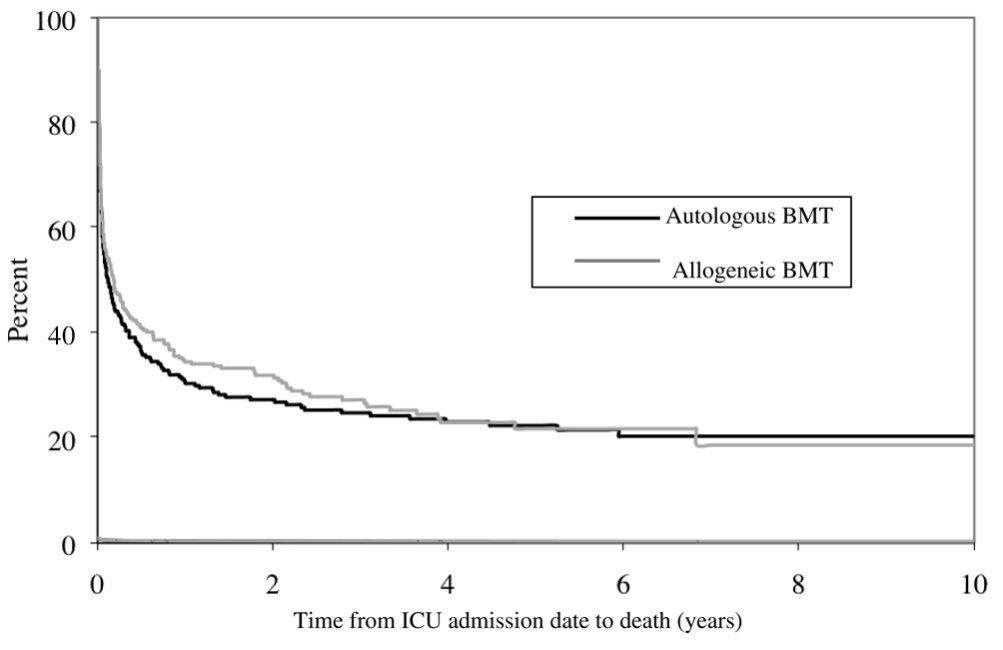
**Survival of bone marrow transplant recipients following intensive care unit admission**. Kaplan–Meier curve showing survival. *y *axis, percentage of patients who required subsequent intensive care unit (ICU) admission following discharge from bone marrow transplant (BMT) (n = 504); *x *axis, time in years from ICU admission during subsequent hospitalization. Black lines, survival following autologous BMT; gray lines, survival following allogeneic BMT.

Most patients died within the first year after ICU admission, whereas survival declined only modestly during subsequent years (Figure 2). Patients receiving any ICU procedure had higher 1-year mortality than those patients not receiving a procedure (87% versus 44%, *P *< 0.0001). Death at 1 year was specifically more frequent if patients required mechanical ventilation (87%; 95% confidence interval, 82% to 91%), pulmonary artery catheterization (91%; 95% confidence interval, 84% to 96%), or hemodialysis (94%; 95% confidence interval, 81% to 99%). Only about 7% of patients who were mechanically ventilated for 10 days or longer survived, and all 16 mechanically ventilated patients who received multiple ICU admissions during the same hospitalization died. Exactly eight patients receiving hemodialysis during their ICU stay required chronic hemodialysis during the subsequent year. The strongest independent predictors of death were mechanical ventilation and hemodialysis (Table 5). Multivariable regression analysis could not be completed because of the small number of survivors in each stratum.

**Table 5:** Outcomes following specific intensive care unit procedures

	Number	Died^a ^(%)	Odds ratio^b ^(95% confidence interval)
Mechanical ventilation alone	258	224 (87)	7.4 (4.8 to 11.4)
Mechanical ventilation > 10 days	67	62 (93)	7.1 (2.8 to 18.0)
Pulmonary artery catheterization	94	86 (91)	6.6 (3.1 to 14.0)
Hemodialysis	35	33 (94)	8.7 (2.1 to 36.7)
Mechanical ventilation and pulmonary artery catheterization	86	79 (92)	6.8 (3.0 to 15.1)
mechanical ventilation and hemodialysis	29	27 (93)	7.0 (1.6 to 29.8)
Pulmonary artery catheterization and hemodialysis	12	11 (92)	5.4 (0.7 to 42.6)

^a^Death within 1 year of intensive care unit (ICU) admission.

^b^Comparison is with all patients without specified procedures or combinations of procedures.

We further examined survivors of multiple procedures during ICU admission. Eight survivors received mechanical ventilation plus either pulmonary artery catheterization or hemodialysis. In this subgroup the median time interval between bone marrow transplant and ICU admission was 1.3 years (interquartile range, 0.36 to 4.5 years). The median ICU length of stay was 6.5 days (interquartile range, 2.0 to 31 days). The survivors' mean age was 45 years (standard deviation, 7.3 years) and most survivors (n = 7) were male.

## Discussion

We studied 2,653 consecutive patients undergoing bone marrow transplant over a decade in Ontario and found that ICU admission during subsequent hospitalizations is associated with high mortality (67%). Mortality rates in this cohort were similar to those observed immediately post-transplant, but attenuated slightly as the time interval since the initial transplant increased. Death rates at 1 year were highest amongst patients requiring aggressive ICU treatments such as mechanical ventilation (87%), pulmonary artery catheterization (91%), and hemodialysis (94%). We could find no combination of ICU procedures that would uniformly predict death, and survival rates did not vary substantially considering multiple procedures.

Outcomes following ICU procedures in our study are similar to those previously reported for the early post-transplant period: the pooled mortality in 21 studies of mechanical ventilation during the early post-transplant period was 90% (1,974 of 2,183 patients; range, 55% to 100%), in four studies of acute renal failure was 78% (104 of 134 patients; range 69% to 100%), and in one study reporting pulmonary artery catheterization was 86% (18 of 21 patients) (Additional File 1). In Ontario, most patients (96%) were admitted to a bone marrow transplant special care unit during this early transplant period. We are unable to determine reasons why 4% of bone marrow transplant recipients were not admitted to these units. Our results show that ICU care continues to be associated with a poor prognosis years after the time of the initial bone marrow transplant.

Investigators have proposed strategies for identifying futile situations for bone marrow transplant recipients on the basis of multiple adverse characteristics [9]. The largest such study observed no survivors amongst 398 patients who had acute lung injury and who had either received more than 4 hours of therapy with vasoactive medications or sustained hepatic and renal failure [30]. We were unable to test this nuance because data about these clinical combinations were not available in this large sample size of patients.

Previous studies documenting 100% mortality in some subgroups have stimulated recommendations to restrict intensive care for selected bone marrow transplant recipients [18,31]. These subgroups tend to be small, however, limiting the precision of survival estimates. Other authors suggest that physicians forego treatment if success is attainable in fewer than one in 100 cases [32]; however, no subgroup in our study fulfilled this quantitative threshold for futility. Examining specific subgroups of bone marrow transplant recipients to identify characteristics that reliably predict survival rates < 1% requires an enormous sample size.

The results from our large study support the concept that ICU care should not be systematically withheld from bone marrow transplant recipients [33]. We detected survivors in each of our prespecified strata, including patients receiving multiple procedures and those requiring prolonged mechanical ventilation. The number needed to treat in the ICU to save one life at 1 year was never greater than 20 for any subgroup. This effect size would be considered adequate to justify inexpensive therapies [34]. Moreover, our study reflects some care from more than a decade ago, suggesting that future technology improvements may make future prognoses even better [19].

Our retrospective study lacks information on several factors that might influence prognosis, such as the specific reason for ICU admission, the degree of acute physiological disturbance, and the use of other ICU procedures or medications. The strength of our study is its large sample size and multicenter recruitment [30]. Our extended observation interval also indicates that advances in the care of bone marrow transplant patients may have improved their prognosis in the community [30,35], yet we detected no such trend in the ICU.

Previous research has reported greater complication rates and mortality with allogeneic bone marrow transplants compared with autologous transplants [36-40]. Contrary to these previous studies, our research does not show a short-term survival benefit with autologous bone marrow transplantation. Instead, the probable explanation for the observed survival differences (Figure 1) is probably different indications and severity of disease, as was typically controlled for in other smaller studies. These nuances may be the core reason why ICU survival and medical futility are so difficult to predict. No standardized admission criteria for bone marrow transplant recipients existed, so our databases cannot explain decisions to provide or withhold ICU admission from specific subgroups of patients (including type of transplant). Finally, our database lacked information regarding other long-term outcomes that might be important to ICU survivors such as the health-related quality of life, functional status, or ongoing care requirements, which remain a topic for future research [41,42].

## Conclusion

Defining futile situations requires a large sample size to establish that good outcomes are sufficiently infrequent. In addition, quantifying such thresholds is somewhat arbitrary and requires consensus amongst stakeholders. The detection of survivors in every stratum of our cohort suggests that ICU care for bone marrow transplant recipients should not be considered futile, contrary to popular opinion or economic incentives. Our results can be used to counsel patients and family members about prognosis and guiding ICU care for bone marrow transplant recipients who consider these therapies to be appropriate.

## Key messages

• The prognosis of bone marrow transplant recipients receiving ICU care during subsequent hospitalizations is very poor but should not be considered futile.

• The strongest independent predictors of death at 1 year in this cohort were mechanical ventilation and hemodialysis.

• No combination of ICU procedures uniformly predicted 100% mortality at 1 year.

## Abbreviations

ICU = intensive care unit.

## Competing interests

The authors declare that they have no competing interests.

## Authors' contributions

DCS, WJS and DAR were responsible for the study concept and design. DCS and DT were responsible for acquisition of the data. DCS and DAR drafted the manuscript. DCS, DT, AK and DAR performed the statistical analyses. DCS, DT, WJS and DAR provided administrative, technical, or material support. DCS and DAR were responsible for study supervision. All authors analyzed and interpreted the data, and critically revised the manuscript for important intellectual content. The principal investigator DCS had full access to all the data in the study and takes responsibility for the integrity of the data and the accuracy of the data analyses.

## Supplementary Material

Additional File 1 Additional file 1 is a Word file containing an evidentiary table summarizing the results of previous studies evaluating outcomes following intensive care unit (ICU) admission for bone marrow transplant recipients. These studies were retrieved using the following Medline (OVID) search strategy: (1) exp/Critical Care; OR exp/Intensive Care; OR exp/Respiration, Artificial; OR exp/Respiratory Distress Syndrome, Adult; OR exp/Multiple Organ Failure; OR exp/Sepsis; OR exp/Sepsis Syndrome; AND (2) exp/Bone Marrow Transplantation; OR exp/Hematopoietic Stem Cell Transplantation. Note that blank fields in the table denote information not contained in the cited publication. sd, standard deviation; IQR, interquartile range; BMT, bone marrow transplant; ARF, acute renal failure. *Acute renal failure defined as doubling of creatinine. ^+^Acute renal failure defined as need for hemodialysis. ^#^Acute renal failure defined as a rise in creatinine. Click here for file
