# Supplementary material for: Intensive care outcomes in bone marrow transplant recipients: a population-based cohort analysis
Source: Crit Care. 2008 Jun 11;12(3):R77. doi: 10.1186/cc6923 (PMC2481474; doi:10.1186/cc6923)
Supplement: Additional File 1 — is a Word file containing an evidentiary table summarizing the results of previous studies evaluating outcomes following intensive care unit (ICU) admission for bone marrow transplant recipients. These studies were retrieved using the following Medline (OVID) search strategy: (1) exp/Critical Care; OR exp/Intensive Care; OR exp/Respiration, Artificial; OR exp/Respiratory Distress Syndrome, Adult; OR exp/Multiple Organ Failure; OR exp/Sepsis; OR exp/Sepsis Syndrome; AND (2) exp/Bone Marrow Transplantation; OR exp/Hematopoietic Stem Cell Transplantation. Note that blank fields in the table denote information not contained in the cited publication. sd, standard deviation; IQR, interquartile range; BMT, bone marrow transplant; ARF, acute renal failure. *Acute renal failure defined as doubling of creatinine. +Acute renal failure defined as need for hemodialysis. #Acute renal failure defined as a rise in creatinine. [file cc6923-S1.doc]

**Additional File: Evidentiary table of intensive care outcomes in bone marrow transplant recipients**

**Table A: Evidentiary Table of Intensive Care Outcomes in Bone Marrow Transplant Recipients**

| Study | Design | BMT Patients | Centres | Study Period | Age | ICU Admission | | Mechanical Ventilation |  | Pulmonary artery catheter | | Acute Renal Failure | | Time between BMT and ICU (days) | Time of Mortality Measurement (days) |
| --- | --- | --- | --- | --- | --- | --- | --- | --- | --- | --- | --- | --- | --- | --- | --- |
|  |  |  |  |  |  | Number (%) | Died (%) | Number (%) | Died (%) | Number (%) | Died (%) | Number (%) | Died (%) |  |  |
| Abraham et al.[1] | Retrospective Cohort | 50 | 1 | Oct 1993 to Mar 1995 |  | 4 (8) | 2 (50) | 3 (75) | 2 (67) |  |  |  |  |  | unknown |
| Afessa et al.[2] | Retrospective Cohort |  | 1 | Aug 1996 to Dec 2000 | mean 49 (sd 13) | 112 | 58 (52) | 62 (55) | 46 (74) |  |  |  |  | median 17 (> 100d in 26 patients) | - 30 |
| Afessa et al.[3] | Retrospective Cohort | 147 | 1 | Apr 1982 to Dec 1990 | mean 36 | 35 (24) | 27 (77) | 27 (77) | 25 (93) | 21 (60) | 18 (86) | 5 (14) | 5 (100) | survivors: mean 247 (sd 158)  nonsurvivors: mean 84 (sd 110) | unknown |
| Bach et al.[4] | Prospective Cohort |  | 5 | Jul 1994 to Jun 1997 | mean 43 (sd 11) |  |  | 226 |  |  |  |  |  | median 27 (range 0-365) | unknown |
| Crawford et al[5]. | Retrospective Cohort | 1482 | 1 | Jan 1986 to Jul 1990 | mean 29 (range 7 to 63) |  |  | 348 | 338 (97) |  |  |  |  | mean 39  (range 0 to 172) | - 180 |
| Ewig et al.[6] | Retrospective Cohort |  | 1 | Jan 1984 to Dec 1993 | mean 36 (sd 15; range 8-76) | 52 | 47 (90) |  |  |  |  |  |  |  | unknown |
| Faber-Langendoen et al. [7] | Retrospective Cohort | 653 | 1 | 13 years |  |  |  | 191 | 185 (97) |  |  |  |  |  | - 180 |
| Gruson et al.[8] | Retrospective Cohort |  |  | Nov 1993 to Dec 1997 | mean 34 (sd 8) | 38 | 29 (76) | 32 |  |  |  |  |  | mean 11 | unknown |
| Hennessy et al.[9] | Retrospective Cohort | 141 | 1 | 1990 to 1997 |  | (15) |  | 21 (15) | 21 (100) |  |  |  |  | median 90 (maximum 17 months) | unknown |
| Huaringa et al.[10] | Retrospective Cohort | 619 | 1 | 1992 to 1993 | median 29 |  |  | 60 | 49 (82) |  |  |  |  |  | unknown |
| Jackson et al.[11] | Retrospective Cohort | 677 | 1 | Jan 1988 to Dec 1993 | median 35 (range 12 to 61) | 116 | 89 (77) | 92 | 76 (83) |  |  | 24 |  | ICU during BMT admission (n=111) | - 180 |
| Khassawneh et al.[12] | Retrospective Cohort | 1301 | 1 | Mar 1991 to Apr 1999 | mean 55 (sd 12) |  |  | 78 | 58 (74) |  |  |  |  | ICU during BMT admission | - hospital |
| Kim et al.[13] | Retrospective Cohort | 210 | 1 | Jan 1999 to Aug 2001 | median 34 (range 19-66) | 18 (9) | 17 (94) |  |  |  |  | 17 (94) |  |  | - hospital |

**Table A (continued)**

| Study | Design | BMT Patients | Centres | Study Period | Age | ICU Admission | | Mechanical Ventilation |  | | Pulmonary artery catheter | | | Acute Renal Failure | | Time between BMT and ICU (days) | | Time of Mortality Measurement (days) |
| --- | --- | --- | --- | --- | --- | --- | --- | --- | --- | --- | --- | --- | --- | --- | --- | --- | --- | --- |
|  |  |  |  |  |  | Number (%) | Died (%) | Number (%) | Died (%) | | Number (%) | | Died (%) | Number (%) | Died (%) |  |  |  |
| Kress et al.[14] | Retrospective Cohort |  | 1 | Jul 1993 to Dec 1996 | median 47 (IQR 36 - 53) | 44 | 17 (39) | 20 (45) | 11 (55) |  | |  | |  |  | |  | - hospital |
| Letourneau et al[15] | Retrospective Cohort | 441 | 1 | Jan1994 to Dec 1998 | mean 44 (sd 11) | 57 (13) |  | 43 (75) |  |  | |  | | 42^*^ (74)  14^+^ (24) | 37^*^ (88) | |  |  |
| Martin et al.[16] | Retrospective Cohort | 350 |  | Jun1981 to Dec 1987 | median 28 (range 1 to 67) |  |  | 24 | 22 (92) |  | |  | |  |  | |  | unknown |
| Paz et al.[17] | Retrospective Cohort | 229 | 1 | Mar 1984 to Mar 1991 | mean 34 (sd 9) | 36 | 24 (67) | 28 (78) | 26 (96) |  | |  | |  |  | |  | unknown |
| Pène et al[18] | Retrospective Cohort | 1025 | 3 | Jan 1997 to Dec 2003 | Median 41 (IQR 29 to 49) | 209 | 165 (79) | 122 (58) | 109 (89) |  | |  | | 59 (29) |  | | 1/3 of patients within 30 days; 2/3 > 30 days | - one-year |
| Price et al.[19] | Prospective Cohort |  | 1 | Jul 1994 to Feb 1996 | median 43 (range 18 - 63) | 115 | 62 (54) | 48 (42) | 39 (81) |  | |  | | 18 (16) | 14 (78) | | ICU during BMT admission | - hospital |
| Rubenfeld & Crawford[20] | Nested Case-Control | 3635 | 1 | Jan 1980 to Jul 1992 |  |  |  | 865 (24) | 812 (94) |  | |  | |  |  | | ICU during BMT admission | - 30 (or hospital discharge) |
| Scott et al[21] | Retrospective Cohort |  | 1 | 1988 to 1998 |  |  |  | 50 | 44 (88) |  | |  | |  |  | |  | - 6 months |
| Shorr et al.[22] | Retrospective Cohort | 159 | 1 | Sep 1990 to Feb 1997 | median 41 (range 19 to 66) | 20 (12) | 14 (70) | 17 | 14 (82) |  | |  | |  |  | | ICU during BMT admission (except 1 patient) | - hospital |
| Soubani et al.[23] | Retrospective Cohort | 745 | 1 | Jan 1998 to Jun 2001 | mean 47 (sd 11) | 85 (11) | 50 (59) | 51 (60) | 32 (63) |  | |  | | 11^+^ (13)  58^#^ (50) | 8^+^ (73)  40^#^ (80) | | ICU during BMT admission  (median 5 weeks; 12 admitted 1 year after transplant) | - hospital  - 6 months in some patients |
| Torrecilla et al.[24] | Retrospective Cohort | 57 | 1 | Mar 1981 Jun 1987 | median 27 (IQR 20 - 37) | 25 (44) | 22 (88) | 16 (64) | 15 (94) |  | |  | |  |  | | range 10 to 350 days | - hospital |

**References**

1. Abraham BB, Hardan I, Segal E, Stemmer SM, Perel A: **Respiratory failure and intensive care treatment in bone marrow-transplanted patients.** *Intensive Care Medicine* 1996, **22**: 269-70.

2. Afessa B, Tefferi A, Dunn WF, Litzow MR, Peters SG: **Intensive care unit support and Acute Physiology and Chronic Health Evaluation III performance in hematopoietic stem cell transplant recipients.[see comment].** *Critical Care Medicine* 2003, **31**:1715-21.

3. Afessa B, Tefferi A, Hoagland HC, Letendre L, Peters SG: **Outcome of recipients of bone marrow transplants who require intensive-care unit support.[see comment].** *Mayo Clinic Proceedings* 1992, **67**: 117-22.

4. Bach PB, Schrag D, Nierman DM, Horak D, White P, Jr., Young JW *et al*.: **Identification of poor prognostic features among patients requiring mechanical ventilation after hematopoietic stem cell transplantation.** *Blood* 2001, **98**: 3234-40.

5. Crawford SW, Petersen FB: **Long-term survival from respiratory failure after marrow transplantation for malignancy.** *American Review of Respiratory Disease* 1992, **145**: 510-4.

6. Ewig S, Torres A, Riquelme R, El Ebiary M, Rovira M, Carreras E *et al*.: **Pulmonary complications in patients with haematological malignancies treated at a respiratory ICU.** *European Respiratory Journal* 1998, **12**: 116-22.

7. Faber-Langendoen K, Caplan AL, McGlave PB: **Survival of adult bone marrow transplant patients receiving mechanical ventilation: a case for restricted use.** *Bone Marrow Transplant* 1993, **12:** 501-507.

8. Gruson D, Hilbert G, Portel L, Boiron JM, Bebear CM, Vargas F *et al*.: **Severe respiratory failure requiring ICU admission in bone marrow transplant recipients.** *European Respiratory Journal* 1999*,* **13**: 883-7.

9. Hennessy BJ, White M, Crotty GM: **Predicting death in mechanically ventilated recipients of bone marrow transplants.** *Annals of Internal Medicine* 1997, **127**: 88.

10. Huaringa AJ, Leyva FJ, Giralt SA, Blanco J, Signes-Costa J, Velarde H *et al*.: **Outcome of bone marrow transplantation patients requiring mechanical ventilation.[see comment].** *Critical Care Medicine* 2000*,* **28**: 1014-7.

11. Jackson SR, Tweeddale MG, Barnett MJ, Spinelli JJ, Sutherland HJ, Reece DE *et al*.: **Admission of bone marrow transplant recipients to the intensive care unit: outcome, survival and prognostic factors.** *Bone Marrow Transplantation* 1998*,* **21**: 697-704.

12. Khassawneh BY, White P, Jr., Anaissie EJ, Barlogie B, Hiller FC: **Outcome from mechanical ventilation after autologous peripheral blood stem cell transplantation.** *Chest* 2002, **121**: 185-8.

13. Kim SW, Kami M, Urahama N, Yamamoto R, Hori A, Imataki O *et al*.: **Feasibility of acute physiology and chronic health evaluation (APACHE) II and III score-based screening in patients receiving allogeneic hematopoietic stem-cell transplantation.** *Transplantation* 2003, **75**: 566-70.

14. Kress JP, Christenson J, Pohlman AS, Linkin DR, Hall JB: **Outcomes of critically ill cancer patients in a university hospital setting.** *American Journal of Respiratory & Critical Care Medicine* 1999*,* **160**: 1957-61.

15. Letourneau I, Dorval M, Belanger R, Legare M, Fortier L, Leblanc M: **Acute renal failure in bone marrow transplant patients admitted to the intensive care unit.** *Nephron* 2002, **90**: 408-12.

16. Martin C, Maraninchi D, Saux P, Blaise D, Blache JL, Gouin F: **Need for mechanical ventilation in the treatment of acute infections complicating bone marrow transplantation.** *Critical Care Medicine* 1990*,* **18**: 120-1.

17. Paz HL, Crilley P, Weinar M, Brodsky I: **Outcome of patients requiring medical ICU admission following bone marrow transplantation.** *Chest* 1993*,* **104**: 527-31.

18. Pene F, Aubron C, Azoulay E, Blot F, Thiery G, Raynard B *et al*.: **Outcome of critically ill allogeneic hematopoietic stem-cell transplantation recipients: a reappraisal of indications for organ failure supports.** *J Clin Oncol* 2006, **24:** 643-649.

19. Price KJ, Thall PF, Kish SK, Shannon VR, Andersson BS: **Prognostic indicators for blood and marrow transplant patients admitted to an intensive care unit.** *American Journal of Respiratory & Critical Care Medicine* 1998, **158**: 876-84.

20. Rubenfeld GD, Crawford SW: **Withdrawing life support from mechanically ventilated recipients of bone marrow transplants: a case for evidence-based guidelines.[see comment].** *Annals of Internal Medicine* 1996*,* **125**: 625-33.

21. Scott PH, Morgan TJ, Durrant S, Boots RJ: **Survival following mechanical ventilation of recipients of bone marrow transplants and peripheral blood stem cell transplants.** *Anaesth Intensive Care* 2002, **30:** 289-294.

22. Shorr AF, Moores LK, Edenfield WJ, Christie RJ, Fitzpatrick TM: **Mechanical ventilation in hematopoietic stem cell transplantation: can We effectively predict outcomes?[see comment].** *Chest* 1999*,* **116**: 1012-8.

23. Soubani AO, Kseibi E, Bander JJ, Klein JL, Khanchandani G, Ahmed HP *et al*.: **Outcome and prognostic factors of hematopoietic stem cell transplantation recipients admitted to a medical ICU. [Review] [34 refs].** *Chest* 2004, **126**: 1604-11.

24. Torrecilla C, Cortes JL, Chamorro C, Rubio JJ, Galdos P, Dominguez d, V: **Prognostic assessment of the acute complications of bone marrow transplantation requiring intensive therapy.** *Intensive Care Medicine* 1988, **14**: 393-8.
